# Massive Radionecrosis vs Pseudoinflammation After Radiosurgical Corpus Callosotomy in a Patient With Drug-Resistant Epilepsy

**DOI:** 10.7759/cureus.91429

**Published:** 2025-09-01

**Authors:** Juan Romero Valencia, Sergio Moreno Jiménez, Guillermo Axayacalt Gutierrez-Aceves, Martha Lilia L Tena Suck, Emilio García Gómez

**Affiliations:** 1 Neurosurgery, Clinical Research, Instituto Nacional de Neurología y Neurocirugía "Manuel Velasco Suarez", México City, MEX; 2 Neurosurgery, Instituto Nacional de Neurología y Neurocirugía Manuel Velasco Suárez, Mexico City, MEX; 3 Neurosurgery, ABC Medical Center, Mexico City, MEX; 4 Radioneurosurgery Department, Instituto Nacional de Neurologia y Neurocirugia, Mexico City, MEX; 5 Neuropathology, Instituto Nacional de Neurología y Neurocirugia, Mexico City, MEX; 6 Epilepsy Clinic, Instituto Nacional de Neurología y Neurocirugía Manuel Velasco Suárez, Mexico City, MEX

**Keywords:** corpus callosotomy, epilepsy, pseudo inflammation, radionecrosis, radiosurgery

## Abstract

Radiosurgery (RS) has proven to be an effective alternative for the treatment of intracranial neoplasms, arteriovenous malformations, and functional conditions such as epilepsy. Regarding the latter, palliative epilepsy surgery involving corpus callosum disconnection by RS has been shown to be as effective as its surgical counterpart in terms of seizure reduction. However, adverse effects derived from radiation-induced neurotoxicity in the target area may occasionally occur, with radionecrosis (RN) being one of the main causes of morbidity after RS. This paper presents the case of a female patient diagnosed with refractory epilepsy who developed an extensive heterogeneous lesion evident on MRI studies months after undergoing callosotomy by RS.

## Introduction

Radiation side effects on the brain can be classified into three categories according to their timing: acute (during or immediately after radiation), subacute or early (usually up to 12 weeks after radiation), and late (months to years after the end of treatment) [1]. Late effects are mainly characterized by leukoencephalopathy and radiation necrosis (RN), the incidence of which depends on factors such as the administered dose, its fractionation, the length of the follow-up period, the location, and the volume treated [2].

RN represents the main limiting toxicity related to radiosurgery (RS). It usually occurs in 5-10% of the treatments performed [2, 3]. RN is believed to be a late complication of radiation, frequently manifesting months or even years after RS ​​treatment (with a median of seven to eight months after RS) [2]. Although its pathophysiological mechanism is not entirely clear, it is believed that radiation necrosis represents inflammation, death, and decomposition of tissue. This is generally associated with surrounding edema, which can be symptomatic or asymptomatic. Symptoms include headache, nausea, vomiting, ataxia, seizures, and functional deficits depending on the location of the focal damage [4].

RN constitutes a clinical challenge due to its potential neurological implications and the negative impact it can have on the patient's quality of life and functional status. For this reason, a timely diagnosis is essential, which must be based on adequate clinical correlation complemented by imaging studies that include specific sequences. Likewise, the early implementation of medical-surgical treatment is crucial to prevent an unfavorable or potentially catastrophic outcome [5, 6]. While RN is a known complication, its diagnosis can become exceptionally challenging when it presents with atypical features that mimic other pathologies. This report illustrates such a diagnostic dilemma through the case of a patient who developed a massive, inflammatory lesion mimicking a neuroinfection following radiosurgical callosotomy.

## Case presentation

We present the case of a patient in her fifth decade of life at the time of her last follow-up, with no significant family history. At nine years of age, she was diagnosed with Lennox-Gastaut syndrome. Later, at age 18, she developed atonic seizures with a frequency of approximately three episodes per day. Ten years later, she underwent open callosotomy.

Due to persistent seizures despite appropriate use of multiple antiseizure medications, she was referred to our institution's epilepsy clinic for treatment of drug-resistant epilepsy (DRE). Following a comprehensive evaluation, the patient was considered a candidate for corpus callosotomy (CC) using stereotactic radiosurgery (SRS).

Pre-treatment evaluation included a high-resolution 3-Tesla brain MRI, which was essential to obtain detailed anatomical delineation of the interhemispheric structures and to accurately define the irradiation target within the anterior portion of the corpus callosum.

**Figure 1:**
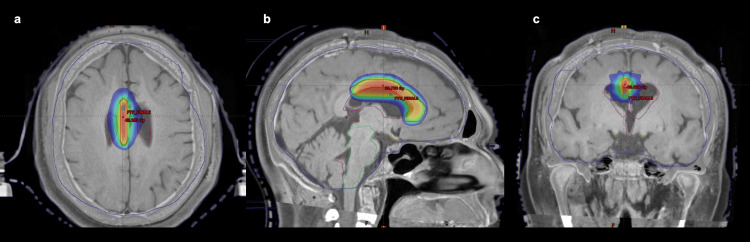
T1-weighted MRI with superimposed isodose lines (a) Axial, (b) sagittal, and (c) coronal T1-weighted MRI with superimposed isodose lines illustrating the radiosurgical dose distribution targeting the anterior corpus callosum. The treatment plan was delivered using six non-coplanar dynamic conformal arcs with a single isocenter. A prescribed peripheral dose of 32 Gy was administered, as shown by the innermost red isodose line encompassing the defined planning target volume (PTV_rCC). The isodose gradient displays a sharp falloff outside the target, confirming the high conformality of the dose distribution and the preservation of surrounding eloquent structures, including the cingulate gyri, ventricles, and adjacent thalamic nuclei.

This imaging protocol allowed for precise volumetric segmentation and integration into the treatment planning system. Following the imaging workup, stereotactic radiosurgical treatment was performed using a frame-based radiation technique. A single high-dose fraction of 32 Gy was prescribed to the periphery of the defined target volume, encompassing the intended callosal segment while minimizing radiation exposure to adjacent critical structures, such as the cingulate gyri and ventricular walls. Treatment delivery was carried out using a TrueBeam® linear accelerator (Varian Medical Systems, Palo Alto, USA) equipped for intracranial stereotactic radiosurgery. The dose was administered through six non-coplanar dynamic conformal arcs, optimized to achieve a highly conformal isodose distribution centered on the anterior midbody of the corpus callosum. The entire procedure was conducted using a single isocenter, allowing for submillimetric accuracy in dose localization. Dosimetric verification and quality assurance protocols were applied before irradiation to ensure safety and reproducibility of the treatment plan (Figure 1).

**Figure 2:**
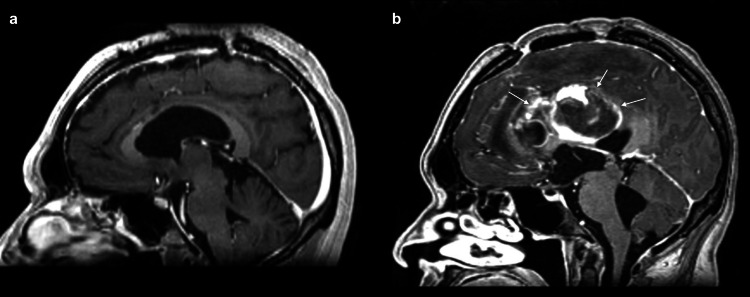
Sagittal T1-weighted brain MRI with gadolinium contrast enhancement obtained at two time points in a patient undergoing stereotactic radiosurgical callosotomy (a) Shows baseline images acquired three months before radiosurgery, demonstrating a corpus callosum without signal abnormalities. (b) Depicts the follow-up MRI obtained 18 months after treatment, revealing focal contrast enhancement and T1 signal hyperintensity within the irradiated segment of the anterior corpus callosum, indicated by the white arrows, consistent with post-radiosurgical changes. These findings suggest localized disruption of white matter integrity in the target region, in accordance with the planned therapeutic goal.

Several weeks later, the patient presented to our center's emergency department with fever, headache, and recent-onset absence seizures. Clinical evaluation revealed signs of intracranial hypertension. Imaging studies showed a large heterogeneous lesion in the midline region adjacent to the corpus callosum and interventricular area, associated with acute obstructive hydrocephalus (Figures 2a, 2b).

An emergency ventriculostomy was performed, with a biopsy of the lesion observed for histopathological analysis. A cerebrospinal fluid (CSF) sample was subsequently obtained, which was culture-negative. Given the patient's favorable clinical progress, the drain was removed, and she was discharged.

One month later, the patient was readmitted to the emergency department with headache, fever, vomiting, chills, and uncontrolled atonic seizures. During her physical evaluation, the patient was observed to be obtunded and had a delayed response to external stimuli. After admission, a lumbar puncture was performed, revealing turbid, brown-colored CSF with the following findings: glucose 11 mg/dL, proteins 842 mg/dL, cell count 1,269/mm³ with 97% mononuclear cells, and lactate 13.7 mmol/L. A positive QuantiFERON® test result confirmed the suspicion of central nervous system tuberculosis.

Empirical antituberculous therapy was initiated, along with vancomycin and ceftriaxone for possible bacterial coinfection. Imaging revealed a lesion involving the septum pellucidum and corpus callosum. Given the clinical context, a tuberculoma was suspected. A second biopsy and complete resection of the mass were performed, and a prophylactic ventriculostomy was placed.

**Figure 3:**
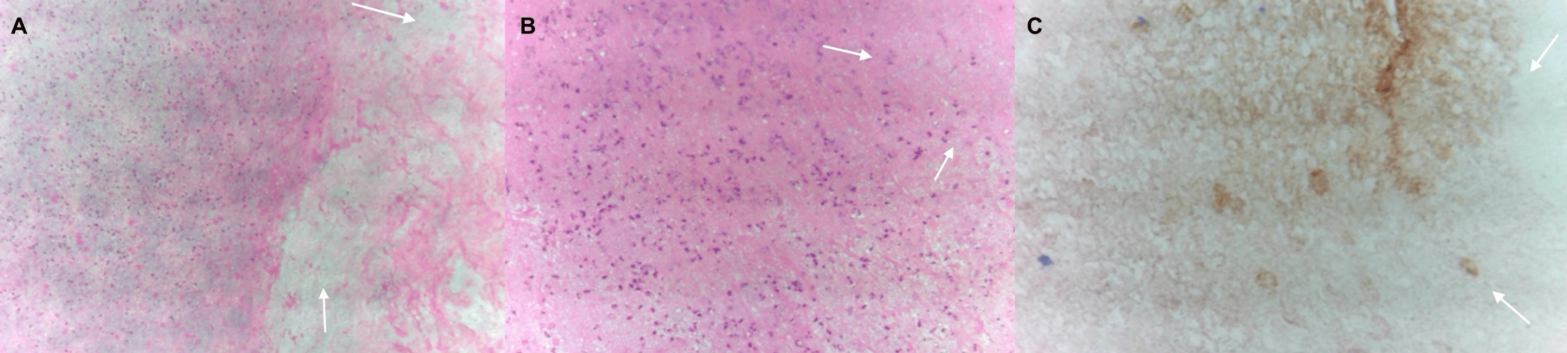
Surgical biopsy specimen In this surgical biopsy specimen, we can observe in the following panels:  A) Brain tissue showing intense necrosis (hematoxylin and eosin x 100) shown by the white arrows. B) Close-up of less necrotic areas showing edema and foci of inflammatory cells (hematoxylin and eosin x 40 ) shown by the white arrows. C) Immunohistochemical (IHQ) staining for Glial Fibrillary Acidic Protein (PGAF).  shows cell ghosts in the extensive areas of necrosis (IHQ x400) shown by the white arrows.

Histopathological analysis revealed extensive coagulative necrosis without findings consistent with tuberculosis-related infection (Figure 3).

Given the favorable postoperative course, the external ventricular drain was removed, and the patient was discharged in stable condition.

## Discussion

RS has proven to be an effective noninvasive therapy for treating various neurological, vascular, oncological, and functional pathologies. This treatment is based on the precise administration of high doses of radiation directed at specific brain areas, with millimeter-level delimitation, which avoids affecting adjacent structures and thus preserves their eloquent function [1]. However, one of the most feared complications is radiation necrosis (RN).

The case presented herein illustrates an unusually aggressive and massive form of RN that initially mimicked infectious or inflammatory pathology, complicating the clinical picture. The lesion developed 18 months post-RS-consistent with late-onset RN-but the presence of fever, turbid cerebrospinal fluid (CSF), and positive QuantiFERON® led to a preliminary suspicion of central nervous system tuberculosis.

Regarding the RN context, MRI typically reveals a ring-enhancing lesion with surrounding edema, which can be indistinguishable from tumor recurrence or other pathologies [6]. This highlights a key clinical dilemma, which is differentiating RN from infectious or neoplastic processes, especially in atypical presentations. Furthermore, the magnitude of the lesion observed on MRI-causing acute hydrocephalus and requiring ventriculostomy highly uncommon in typical RN cases. Moreover, the CSF profile resembled bacterial or tuberculous meningitis, emphasizing the importance of integrating radiological, clinical, and histopathological data to reach a correct diagnosis [6].

 The mechanisms of radiation on the brain have been widely studied [1, 7, 8]. These include neuronal apoptosis, vascular necrosis, and, more recently, the neuromodulatory effects and reorganization of neuronal synapses described in functional disorders [9, 10]. However, RN is the main adverse effect in terms of limiting the toxicity of RS, occurring in approximately 5 to 10% of treated cases, and its incidence generally increases with higher radiation doses, larger fraction sizes, and the administration of chemotherapy [1, 8]. The radiation tolerance of normal tissues depends on several factors, including total dose, dose per fraction, total exposure time, target volume, radiation quality, and adjunctive therapies. According to the Radiation Therapy Oncology Group, the recommended maximal tolerated dose is 15 Gy for targets measuring 31-40 mm in diameter, 18 Gy for targets between 21-30 mm, and greater than 24 Gy for targets smaller than 20 mm [11, 12].

RN diagnosis can be sometimes challenging. Conventional MRI with contrast-enhanced T1 and T2-fluid-attenuated inversion recovery (FLAIR) remains the first-line tool, though its specificity is limited; functional imaging modalities are particularly valuable when standard MRI is inconclusive [13]. Advanced techniques like MR spectroscopy, perfusion-weighted imaging, and amino acid positron emission tomography (PET) improve diagnostic accuracy by revealing metabolic and perfusion differences between radionecrosis and tumor recurrence. The integration of multiparametric MRI with amino acid PET imaging is currently considered the most effective non-invasive strategy for differentiating RN from tumor recurrence. This multimodal approach improves diagnostic confidence, informs treatment planning, and may prevent unnecessary surgical biopsies [14].

Histologically, vascular abnormalities and demyelination are the predominant features of radiation-induced injury in the central nervous system [15]. Histopathological analysis ultimately demonstrated extensive coagulative necrosis without granulomatous inflammation or infectious organisms, confirming RN. While a biopsy is rarely performed to confirm suspected radionecrosis, it played a crucial role in our case due to uncertainty regarding the underlying etiology.

Although the exact pathogenesis of radionecrosis remains unclear, two main hypotheses have been proposed: vascular injury leading to microvasculopathy and necrosis via transforming growth factor β (TGF-β)-mediated pathways [16-18], and glial damage involving oligodendrocyte apoptosis and proinflammatory cytokines.

Management of RN depends on symptom severity. While corticosteroids are first-line treatment, surgical resection is indicated in refractory cases or when mass effect leads to intracranial hypertension, as seen in our patient. The case also illustrates that even when initial CSF studies and imaging suggest infection, a high index of suspicion for RN must be maintained post-RS, particularly when imaging shows lesions centered on the irradiated volume [3]. 

The most remarkable feature of this case is not the occurrence of radionecrosis itself, but rather its unusually aggressive presentation. The development of a massive lesion causing obstructive hydrocephalus, coupled with systemic symptoms like fever and inflammatory CSF markers, is highly atypical for RN. This mimicry of a neuroinfectious process, further confounded by a positive QuantiFERON® test, created a significant diagnostic pitfall. This highlights the necessity of maintaining RN high on the differential diagnosis for any new lesion in a post-radiosurgery field, regardless of the clinical presentation

## Conclusions

Radionecrosis must be considered in any patient presenting with a new intracranial lesion after radiosurgery, even in the face of atypical clinical and laboratory findings suggesting an infectious etiology. While advanced imaging techniques are valuable, this case demonstrates that in situations of high diagnostic uncertainty, histopathological analysis remains the gold standard. It is crucial for confirming the diagnosis, guiding appropriate management, and preventing unnecessary treatments.

## References

[1] Zaer H, Glud AN, Schneider BM (2020). Radionecrosis and cellular changes in small volume stereotactic brain radiosurgery in a porcine model. Sci Rep.

[2] Miller JA, Bennett EE, Xiao R (2016). Association between radiation necrosis and tumor biology after stereotactic radiosurgery for brain metastasis. Int J Radiat Oncol Biol Phys.

[3] Vellayappan B, Lim-Fat MJ, Kotecha R (2024). A systematic review informing the management of symptomatic brain radiation necrosis after stereotactic radiosurgery and international stereotactic radiosurgery society recommendations. Int J Radiat Oncol Biol Phys.

[4] Milano MT, Grimm J, Niemierko A (2021). Single- and multifraction stereotactic radiosurgery dose/volume tolerances of the brain. Int J Radiat Oncol Biol Phys.

[5] Chung C, Bryant A, Brown PD (2018). Interventions for the treatment of brain radionecrosis after radiotherapy or radiosurgery. Cochrane Database Syst Rev.

[6] Al-Rubaiey S, Senger C, Bukatz J (2024). Determinants of cerebral radionecrosis in animal models: A systematic review. Radiother Oncol.

[7] Nichelli L, Casagranda S (2021). Current emerging MRI tools for radionecrosis and pseudoprogression diagnosis. Curr Opin Oncol.

[8] Lawrence YR, Li XA, el Naqa I, Hahn CA, Marks LB, Merchant TE, Dicker AP (2010). Radiation dose-volume effects in the brain. Int J Radiat Oncol Biol Phys.

[9] Tetzlaff SK, Reyhan E, Layer N (2025). Characterizing and targeting glioblastoma neuron-tumor networks with retrograde tracing. Cell.

[10] Régis J, Bartolomei F, Hayashi M, Chauvel P (2002). Gamma Knife surgery, a neuromodulation therapy in epilepsy surgery!. Acta Neurochir Suppl.

[11] Chao ST, Ahluwalia MS, Barnett GH (2013). Challenges with the diagnosis and treatment of cerebral radiation necrosis. Int J Radiat Oncol Biol Phys.

[12] Chu HH, Choi SH, Ryoo I (2013). Differentiation of true progression from pseudoprogression in glioblastoma treated with radiation therapy and concomitant temozolomide: comparison study of standard and high-b-value diffusion-weighted imaging. Radiology.

[13] Zakhari N, Taccone MS, Torres C (2018). Diagnostic accuracy of centrally restricted diffusion in the differentiation of treatment-related necrosis from tumor recurrence in high-grade gliomas. AJNR Am J Neuroradiol.

[14] Galldiks N, Lohmann P, Fink GR, Langen KJ (2023). Amino acid PET in neurooncology. J Nucl Med.

[15] New P (2001). Radiation injury to the nervous system. Curr Opin Neurol.

[16] Yoshii Y (2008). Pathological review of late cerebral radionecrosis. Brain Tumor Pathol.

[17] Milker-Zabel S, Debus J, C Thilmann, Schlegel W, Wannenmacher M (2001). Fractionated stereotactically guided radiotherapy and radiosurgery in the treatment of functional and nonfunctional adenomas of the pituitary gland. Int J Radiat Oncol Biol Phys.

[18] Yang L, Yang J, Li G, Li Y, Wu R, Cheng J, Tang Y (2017). Pathophysiological responses in rat and mouse models of radiation-induced brain injury. Mol Neurobiol.

